# Prevalence, awareness, medication, control, and risk factors associated with hypertension in Yi ethnic group aged 50 years and over in rural China: the Yunnan minority eye study

**DOI:** 10.1186/s12889-015-1687-4

**Published:** 2015-04-15

**Authors:** Lixing Chen, Yuan Zong, Tao Wei, Xun Sheng, Wei Shen, Jun Li, Zhiqiang Niu, Hua Zhou, Yang Zhang, Yuansheng Yuan, Qin Chen, Hua Zhong

**Affiliations:** The First Affiliated Hospital of Kunming, Medical University, Kunming, China; The First Affiliated Hospital of Nanjing, Medical University, Nanjing, China; Kunming Medical University, Kunming, China; The Second People’s Hospital of Yunnan Province, Kunming, China

**Keywords:** Hypertension, Prevalence, Awareness, Medication, Control, Risk factors, Yi ethnic group

## Abstract

**Background:**

Hypertension is an important public health issue in China, but there are few studies examining hypertension in ethnic groups in Yunnan, China. This study, Yunnan Minority Eye Study (YMES), was initially designed to determine the prevalence and impact of eye diseases, including hypertension and diabetes mellitus. As a part of YMES, the prevalence, awareness, treatment, and control of hypertension and the associated risk factors among the Yi ethnic population in rural China are reported.

**Methods:**

A population-based survey was conducted in 2012 with adult participants over 50 from rural communities in Shilin Yi Autonomous County, Yunnan Province, located in southwest China. A random cluster sampling method was used to select a representative sample. The participants’ blood pressure, height, weight, and waist circumference were measured. Hypertension was defined as mean systolic blood pressure ≥140 mmHg and/or diastolic blood pressure ≥90 mmHg, and/or current use of antihypertensive medications.

**Results:**

A total of 2208 adults were assessed. The prevalence of hypertension was 38.5%, and the age- and gender-adjusted prevalence was 37.0%. The proportion of patients who were aware of their hypertension among those diagnosed with hypertension was 24.8%. Of those aware of having hypertension, 23.6% took antihypertensive drugs. Among all hypertensive patients, only 7.2% had controlled their hypertension (<140/90 mmHg). Risk factors for hypertension were older age, smoking, alcohol consumption, family history of high blood pressure, overweight, and obesity. Protective factors included being slim and higher education.

**Conclusions:**

Hypertension was highly prevalent among this population of the Yi ethnic group in China. The ratio of awareness, treatment, and control of hypertension were considerately low. Hypertension education and screening programs in rural China are recommended to improve the health status of this population.

## Background

Hypertension is a worldwide public health concern due to its high prevalence and concomitant risks of cardiovascular and kidney disease [1]. Hypertension contributes to half of the coronary heart disease and approximately two-thirds of the cerebrovascular disease burdens [2].

China is the world’s largest developing and most populous country, with a population of about 1.35 billion. According to the China National Nutrition and Health Survey 2002, the prevalence of hypertension was 20% in men and 17% in women [3]. In addition, of the 24% of hypertensive participants who were aware of their condition, 78% were treated and 19% were adequately controlled [3]. A survey in 2014 reported the prevalence of hypertension was 31.2% in men and 28.0% in women. Awareness of the condition, treatment among all hypertensive patients, and control of the condition were 42.6%, 34.1%, and 27.4%, respectively [4]. More importantly, the prevalence of hypertension has increased dramatically in the past few decades. More than half of the nantional population lives in rural regions. Previous studies have reported that the prevalence of hypertension in rural areas in 1991, 2002, and 2007 was 20.4%, 24.5%, and 30.6%, respectively [5]. This increasing trend in prevalence is associated with heavy economic burden in treatment and disease prevention.

Only a few studies examining hypertension in ethnic groups in Yunnan, China, which has borders with three countries, Myanmar, Laos, and Viet Nam, have been conducted [6]. Data on hypertension-related issues among the Yi ethnic group are not available. The Yi ethnic minority represents the sixth largest of 55 ethnic minority groups in China. They have their own script and language, which belongs to the Tibeto-Burman language group of the Chinese-Tibetan language family and includes six dialects [7]. Shilin is an Autonomous Prefecture inhabited by the Yi ethnic group. As the area has a mild climate and fertile land, the Yi people are mainly involved with agriculture. They like eating pickled food and peculiar cheese [8]. Yi people have their own special genetic origin, inhabited environment, and customs.

In this study, we aim to determine the prevalence, awareness, medication, and control of hypertension among the Yi ethnic group in Shilin county of southwest China.

## Methods

### Ethical considerations

Informed consent was obtained from all the patients before enrollment in the study. Ethical approval was granted by the Kunming Medical University Ethics Review Board. The study was conducted in accordance with the tenets of the World Medical Association’s Declaration of Helsinki.

### Study participants

Study participants were recruited from a random sample of individuals in Shilin, which is autonomous county inhabited by the Yi ethnic group. Shilin was chosen for the survey as the majority of Yi nationals in China live in this area and its socioeconomic profile is representative of the Yi nationality as a whole.

### Study design

The sampling frame was constructed using geographically defined clusters based on village registry data. Cluster boundaries were defined so that each cluster would have a population of approximately 1000 individuals of all ages. Cluster sampling was used to divide Shilin county into 235 areas. Sample size was based on estimating an anticipated 3.5% prevalence for glaucoma with 95% confidence intervals (95% CI). Because the prevalence of hypertension was much higher than glaucoma in previous studies [9], the sample size was considered adequate. Assuming an examination response rate of 80% and a design effect of 1.25 to account for inefficiencies associated with the cluster sampling design, a sample of 2118 people ≥50 years of age was required. Depending on the percentage of the population ≥50 years of age, 12 to 14 clusters were randomly selected (with equal probability). In present study, there were 33 villages (including 77 sampling clusters) could be the candidates for sampling, and 14 clusters were selected randomly. There were 12857 people were in the selected villages, involved 2732 persons aged 50 years and above (21.2%).

Fieldwork was carried out over a 4-month period beginning in July 2012. Listings of households with the names of residents ≥50 years of age were obtained from village registers, followed by door-to-door household visits conducted by enumeration teams. Those people ≥50 years of age were enumerated by name, gender, age, and education. Individuals temporarily absent at the time of the household visit were included in the enumeration. Unregistered adults ≥50 years of age were enumerated and included in the study sample if they had been living in the household for 6 months or more.

Examination sites were set up at local community facilities within 15 min walking distance for most participants. Study participants were examined on a prescheduled date established at the time of enumeration. The identities of the participants were verified using the participants’ official photo identity cards. Those who did not appear at the examination site were revisited by a member of the enumeration team to encourage participation. Details of the study design, sampling plan, and baseline data are reported elsewhere [9-11].

All study investigators and staff successfully completed a training program that oriented them both to the aims of the study and to the specific tools and methodologies employed. At the training sessions, interviewers were given detailed instructions on administration of the study questionnaire. Data collection was conducted by trained research staff who handed out a standard questionnaire. Information on demographic characteristics (such as age, gender, etc.), hypertension history, general medical history, alcohol consumption, cigarette smoking, and education level was obtained. The interview included questions related to the diagnosis, awareness, and treatment of hypertension.

### Definitions of variables

We revised the categories of cigarette smoking (“occasional smoking” was merged into “no smoking”; and “quitting smoking” into smoking) and education level (“junior college” and “graduate or above” were merged into “junior college and above”) because there were few participants belonging to the abovementioned categories. Drinking was defined as alcohol consumption of 8 g/week or more [12]. Family history of hypertension was defined as a diagnosis of hypertension in one parent. Body weight and height were measured with participants wearing light clothing and without shoes, and body mass index (BMI) was calculated as weight in kilograms divided by height in meters squared. Slim, average weight, overweight, and obese were defined as a BMI <18.50, 18.5–24.99, 25.00–29.99, and ≥30.00 kg/m^2^, respectively [13].

A trained and certified observer used American Heart Association protocol to perform three blood pressure measurements with the participant in the sitting position after 5 min of rest. Participants were advised to avoid alcohol consumption, cigarette smoking, coffee or tea, and to avoid exercise for at least 30 min before these measurements. The research staff used a standardized mercuric-column sphygmomanometer and one of four cuff sizes (pediatric, regular adult, large, or thigh) based on the circumference of the participant’s arm.

The hypertension status was assessed based on the US Seventh Joint National Committee report on the prevention, detection, evaluation, and treatment of high blood pressure [14]. Hypertension was defined as an average systolic blood pressure (SBP) at least 140 mmHg, or an average diastolic blood pressure (DBP) at least 90 mmHg. Patients who self-reported current treatment for hypertension with antihypertensive drug within 2 weeks prior to the interview were also classified as hypertensive patients. This definition excluded hypertensive patients whose blood pressure had been reduced to a nonhypertensive range solely by the use of nonpharmacological measures. Prehypertension was defined as either SBP of 120–139 mmHg or DBP of 80–89 mmHg. The awareness of hypertension was defined by a self-report of a prior diagnosis of hypertension by a healthcare professional. The treatment of hypertension was defined as the self-reported use of pharmacological medication for the management of high blood pressure (HBP) within the 2 weeks preceding the participant’s interview. The control of hypertension was defined as the pharmacological treatment of hypertension with an average SBP <140 mmHg and an average DBP <90 mmHg.

### Statistical analysis

Continuous variables are given as mean ± SD and categorical variables as the percentage in each subgroup. Continuous variables between two groups were compared using the *t*-test, and those among three groups or more using one-way ANOVA. Associations between categorical variables were tested using contingency tables and the Chi-square test. We calculated adjusted odds ratios (OR) with 95% CI for hypertension status using multivariate logistic regression models. All data analyses were conducted using SAS software (Version 9.1; SAS Institute Inc., Cary, NC, USA). All statistical tests were two-tailed, and a p-value <0.05 was considered statistically significant.

## Results

### Prevalence of hypertension

The prevalence of hypertension in the Yi ethnic population aged ≥50 in Yunnan Province was 38.5% (850/2208; 95% CI: 36.5–40.5) for both sexes, 37.7% (358/950; 95% CI: 34.6–40.8) for men, and 39.1% (492/1258; 95% CI: 36.4–41.8) for women. There was no statistically significant difference between men and women (p > 0.05). After adjusting for age and sex, prevalence of hypertension was 37.0% (Table 1).

**Table 1:** **Prevalence of hypertension among Yi people aged 50 or more in Shilin, southwest China, 2012**

**Sex**	**No. of participants**	**No. of participants detected as hypertension**	**Prevalence (95% CI)**
Male	950	358	37.7% (34.6- 40.8%)
Female	1258	492	39.1% (36.4- 41.8%)
Total	2208	850	38.5% (36.5- 40.5%)

### Distribution of hypertension

The US Seventh Joint National Committee report defines prehypertension as either SBP 120–139 mmHg or DBP 80–89 mmHg [14]. The prevalence of prehypertension was 40.6% for both sexes, 42.1% for men, and 37.2% for women (Table 2). Only 20.2% of men and 23.7% women had optimal blood pressure. The prevalence of hypertension stages 1, 2, and 3 were 18.7, 11.2, and 7.8% in men and 19.6, 11.1, and 8.6% in women, respectively.

**Table 2:** **Percentage (%) distribution of blood pressure levels in a rural population of Yi ethnic group in southwest China, 2012**

		**Normotensive or controlled hypertensive**			Hypertensive*		
**Age group (yrs)**	**n**	**Optimal**	**Normal**	**High normal**	**Stage 1**	**Stage 2**	**Stage 3**
SBP		<120	120-129	130-139	140-159	160-179	≥180
DBP		<80	80-84	85-89	90-99	100-109	≥110
Male (total)	950	20.2	19.6	22.5.	18.7	11.2	7.8
50-59	266	25.6	24.2	17.1	16.4	10.2	6.5
60-69	362	24.1.	18.9	23.4	17.2	10.8	7.6
70-79	243	13.5	16.3	26.6	20.4	13.8	9.4
80-	79	20.3	18.6	18.1	20.2	14.6	8.2
Female (total)	1258	23.7	17.2	20.0	19.6	11.1	8.6
50-59	393	27.2	25.4	15.1	16.6	10.4	5.3
60-69	458	20.6	18.8	21.3	18.9	11.2	9.2
70-79	332	15.5.	17.2	24.5	20.1	12.7	10.0
80-	75	18.9	12.7	16.4	25.4	17.2	9.4

*For hypertensive, stage 1 refers to SBP between 140 and 159 mmHg, DBP between 90 and 99 mmHg; stage 2 refers to SBP between 160 and 179 mmHg, DBP between 100 and109 mmHg; and stage 3 refers to SBP ≥ 180 mmHg, DBP ≥ 110 mmHg.

SBP readings for males (142.24 ± 26.09 mmHg) and females (143.58 ± 25.90 mmHg) were not statistically different (p > 0.05). However, DBP readings for males (79.34 ± 14.02 mmHg) was greater than that for females (78.00 ± 13.70 mmHg) (p < 0.05) (Table 3).

**Table 3:** **Blood pressure levels (mean ± SD) in a rural population of Yi ethnic group in southwest China, 2012**

**Age group (yrs)**	**Variable (mmHg)**	**Men (n = 950)**	**Women (n = 1258)**	**T value**	**P value**
50-59	SBP	138.57 ± 23.58	139.41 ± 24.26	1.118	0.264
	DBP	82.05 ± 13.25	76.39 ± 13.63	1.545	0.123
60-69	SBP	143.79 ± 26.21	144.92 ± 26.18	1.390	0.165
	DBP	79.31 ± 14.37	78.01 ± 13.28	1.338	0.181
70-79	SBP	145.67 ± 27.01	149.46 ± 18.00	0.464	0.643
	DBP	86.12 ± 12.14	84.98 ± 13.57	0.687	0.493
80-	SBP	147.82 ± 28.35	148.26 ± 23.15	0.358	0.721
	DBP	83.32 ± 15.26	78.62 ± 12.66	1.441	0.154
Total	SBP	142.24 ± 26.09	143.58 ± 25.90	1.201	0.230
	DBP	79.34 ± 14.02	78.00 ± 13.70	2.263	0.024

### Awareness, treatment, and control of hypertension

The rates of awareness among those diagnosed with hypertension were 24.8% (211/850; 95% CI: 21.9–27.7) for both sexes, 26.0% (93/358; 95% CI: 21.4–30.5) for men, and 24.0% (118/492; 95% CI: 20.2–27.8) for women (Table 4). Of those aware of having hypertension, 23.6% (201/850; 95% CI: 20.3–27.5) took antihypertensive drugs, and 26.1% (61/234; 95% CI: 20.4–31.7) of those treated became normotensive. Among all hypertensive patients in the study, only 7.2% (61/850) had their hypertension under control. No statistically significant differences were observed in awareness and control by gender or by age group (Chi-square p > 0.05). For individuals aged ≥50, the proportions of patients taking antihypertensive medications among those aware of their hypertension decreased with age.

**Table 4:** **Percentage (%) of awareness, treatment and control of hypertension by socio-demographics and those with and without risk factors**

**Variables**	**Hypertension**	**Awareness**	**Treatment**	**Control**
	**(n = 2208)**	**(n = 850)** ^**a**^	**(n = 850)** ^**b**^	**(n = 234)** ^**c**^
Age groups (yrs): 50–59 (reference)	32.8	30.1	32.4	20.0
60-69	38.2*	25.2	28.1*	27.3
70-79	43.1*	21.4	26.2*	29.2
80-	47.4*	19.2	15.1*	36.4
Gender: Men (reference)	37.7	26.0	24.8	29.0
Women	39.1	24.0	22.1	22.3
Education level: No school (reference)	42.8	19.1	24.2	22.9
Primary school	36.4*	29.9*	28.0*	34.1
Junior high school	34.0*	31.2*	32.1*	25.7
Senior high school	31.2*	36.1*	38.6*	25.0
Junior college and above	25.5*	53.8*	38.5*	40.0
Body weight: normal (reference)	39.5	25.0	27.6	22.2
Leanness	31.4*	14.9*	21.4*	32.6
Overweight	54.3*	41.1*	37.9*	30.6
Obesity	65.5*	52.6*	42.1*	37.5
Cigarette smoking: no (reference)	35.4	21.6	26.6	26.3
Smoking	44.0*	29.4*	28.9	25.7
Alcohol consumption: no (reference)	34.9	21.3	26.4	23.2
Yes	45.7*	30.2*	29.3	28.1
Family history of HBP: no (reference)	35.0	23.2	27.1	24.4
Yes	70.4*	32.2*	29.6	26.5

^a^Among participants with hypertension, ^b^Among participants aware of their hypertension diagnosis, ^c^Among participants taking medications for their hypertension. *P < 0.01 in cigarette smoking group, alcohol consumption group, P < 0.05 in age group, education level group, Family history of HBP group (compared with the reference by Chi-square test).

### Risk factors of hypertension

According to the results of the Chi-square test (Table 4), factors which modified the percentage of hypertension in the participants included age, BMI, education level, cigarette smoking, alcohol consumption, and family history of hypertension. For individuals aged ≥50, the percentages of the hypertensive participants who were aware of their hypertension among those diagnosed with hypertension decreased with age. Compared with average weight and slim participants, patients who were overweight or obese were more likely to be aware of hypertension. With an increase in education level, the proportions of awareness and treatment showed an increasing trends. Participants with a family history of hypertension had a greater chance of knowing about the condition compared with those without. The ratio of awareness among these who smoked or drank alcohol were higher than those who did not smoke or did not drink.

To further analyze risk factors of hypertension, hypertension was used as the dependent variable, while age, BMI, education level, cigarette smoking, alcohol consumption, and family history of HBP as independent variables. Multivariate logistic regression models were performed (Table 5), with adjustment for age as well as for all other variables. Participants in the higher age groups (60–69, 70–79, and 80+) appeared to have a higher risk of developing hypertension (OR > 1.0) compared with the 50–59 age group. People who were overweight or obese appeared to have a higher chance of developing hypertension than average weight participants (OR > 1.0), whereas slim participants were less likely to have hypertension (OR < 1.0). Participants with a family history of hypertension or those who drank or smoked had a higher chance of developing hypertension compared with those without a family history of hypertension or who did not drink or smoke. Subjects who finished senior high school, junior college or above had a decreased risk of hypertension (OR < 1.0) compared with those without the levels of of education mentioned above.

**Table 5:** **Factors associated with the prevalence of hypertension from multivariate logistic regression models (N = 2208)**

**Variables**	**n**	**Prevalence of HBP (%)**	**OR (95% CI)** ^**a**^	**OR (95% CI)** ^**b**^
Age groups (years): 50–59	659	32.8	-	1.000(reference)
60-69	820	38.2*	-	1.050(0.724, 1.524)
70-79	575	43.1*	-	2.768(1.728, 4.433)
80-	154	47.4*	-	3.104(1.350, 7.135)
Education level: No school	1139	42.8	1.000(reference)	1.000(reference)
Primary school	431	36.4*	0.703(0.551, 0.897)	0.771(0.600, 0.991)
Junior high school	318	34.0*	0.609(0.460, 0.805)	0.688(0.515, 0.918)
Senior high school	269	31.2*	0.536(0.393, 0.729)	0.642(0.464, 0.889)
Junior college and above	51	25.5*	0.416(0.214,0.811)	0.490(0.251,0.959)
Body weight: normal weight	1323	39.5	1.000(reference))	1.000(reference)
Leanness	685	31.4*	0.669(0.546, 0.821)	0.642(0.522, 0.789)
Overweight	175	54.3*	1.811(1.295, 2.533)	1.897(1.353, 2.662)
Obesity	25	65.5*	3.067(1.385, 6.792)	3.009(1.355, 6.678)
Cigarette smoking: no	1412	35.4	1.000(reference)	1.000(reference)
Yes	796	44.0*	1.745(1.332, 2.287)	1.768(1.348, 2.319)
Alcohol consumption: no	1468	34.9	1.000(reference)	1.000(reference)
Yes	740	45.7*	1.745(1.356, 2.245)	1.788(1.389, 2.303)
Family history of HBP: no	1992	35.0	1.000(reference)	1.000(reference)
Yes	216	70.4*	3.968(2.892,5.445)	3.817(2.776,5.248)

95% CI, 95% confidence interval; HBP, high blood pressure; OR, odds ratio; ^a^Adjusted for age only; ^b^Adjusted for all other variables in the table. *P < 0.01 in cigarette smoking group, alcohol consumption group, P < 0.05 in age group, education level group, Family history of HBP group.

## Discussion

We found an overall prevalence of 38.5% for hypertension among the Yi ethnic group aged ≥50 in Yunnan Province, 37.7% in men and 39.1% in women. The prevalence rate of hypertension in our study was higher than those among people from other areas of China [3,5,15,16], Europe [17], or USA [18,19]. In a sample of Chinese adults aged ≥18, the prevalence of hypertension in 2014 was 29.6% [4]. A hypertension survey conducted between 2001 and 2003 in European population aged from26 to 65 years old indicated that the prevalence of hypertension was 24.4% [17]. However, after adjusting for age (aged ≥ 45), the hypertension prevalence rates in many studies were obviously higher. The prevalence rates of hypertension in rural southeast China (aged ≥50) [20], Thailand (aged ≥60) [21] and New York City (aged ≥ 45) [18] was 54.6%, 51.1%, 40.6%, respectively. The prevalence of hypertension in our study was similar to the prevalence among people from rural southwest China (aged ≥50) [22] and New York City (aged ≥ 45) [18]. It was lower than the prevalence rates in population from Thailand (aged ≥60) [21] and Rural Southeast China (aged ≥50) [20] (Table 6). It appears that age is very important risk factor of hypertension. The prevalence of hypertension is higher by ages.

**Table 6:** **Comparison of prevalence, awareness, treatment, and control of hypertension in China and other countries**

**Study (author/s)**	**Project site**	**Sample (n)**	**Year of study**	**Age**	**Hypertension**			
					**Prevalence**	**Awareness**	**Treatment**	**Control**
The present study	Rural Southwest China	2,208	2012	≥50	37%	24.8%	78.1%^a^ (27.5%^b^)	26.1%^c^ (7.2%^d^)
Wu Y et al. [3]	Rural and urban China	141,892	2002	≥18	18%	25%	80%^a^ (20%^b^)	24%^c^ (5%^d^)
Zhang J et al. [22]	Rural Southwest China	2133	2010	≥50	40.0%	28.4%	86.7%^a^ (24.6%^b^)	30.0%^c^ (7.5%^d^)
Meng XJ et al. [16]	Urban Northeast China	25,196 (n = 4825)	2010	18-74 (≥45)#	28.7% 47.5%#	42.9% 48.1%#	65.7% (28.2%^b^) 70.3%^a^ (27.4%^b^) #	12.9%^c^(3.7%^d^) 12.1%^c^ (4.7%^d^) #
Huang J et al. [20]	Rural Southeast China	5,350	*	0-100 (≥ 50)#	36.09% 54.6%#	28.85%*	*	*
Porapakkham Y et al. [21]	Thailand	19,374	2004	≥60	51.1%	43.9%	36.1%^a^ (8.5%^b^)	10.6%^c^ (1.7%^d^)
Angell SY et al. [18]	New York City, USA	1,975 (n = 787)	2004	≥20 (≥45)#	25.6% 40.6%#	75% 85.7%#	62.5% 75.0%^a^ #	43.6%48.8%^c^ #
Wang J et al. [4]	Chinese adults	50,171	2014	≥18 (≥60)#	29.6% 58.2%#	42.6% 62%#	34.1%^b^ 54.5%^b^#	27.4%^c^ (9.3%^d^) 27.8%^c^ (14.8%^d^) #
Burt VL et al. [19]	USA	9,901	1988-1991	≥18	24%	69%	53%^a^	45%^c^ (24%^d^)
Costanzo S et al. [17]	London (England)	1,604	2001	26-65	20.8%	44%	32.1%^a^	47.7%^c^
	Limburg (Belgium)		2002		23.6%		42.5%^a^	43%^c^
	Abruzzo (Italy)		2003		28.8%		43.5%^a^	42%^c^
Yang J et al. [5]	Rural	8,359	1991	35-74	20.4%	15.0%	8.9%^b^	3.3%^d^
	Eastern	18,922	2002		24.5%	49.0%	38.8%^b^	6.3%^d^
	China	20,167	2007		30.6%	52.4%	38.3%^b^	7.2%^d^

^a^The proportion of hypertensive patients who took antihypertensive medications among hypertensive patients aware of their conditions.

^b^The proportion of hypertensive patients who took antihypertensive medications among all the hypertensive patients.

^c^The proportion of hypertensive patients who had their HBP controlled among hypertensive patients receiving medical treatment for hypertension.

^d^The proportion of hypertensive patients who had their HBP controlled among all the hypertensive patients.

*Denotes the information was not available.

#Adjusted for age (45+).

Yi people have their own special ethnic origin, inhabited environment, and customs. Most of them lived in Shilin, which has a mild climate and fertile land. The Yi people are mainly engaged in agriculture, which leads to low the proportion of overweight and obesity among them. Overweight and obesity were the risk factors of hypertension. However, the prevalence rates of hypertension in Yi group was high. Some possible reasons were as follows. According to the 2013 AHA/ACC Guideline on Lifestyle Management to Reduce Cardiovascular Risk, consuming no more than 2,400 mg of sodium per day can result in a reduction in blood pressure [23]. Of the hypertensive patients in the current study, 79.1% (1746/2208) of Yi people reported a preference for preserved food, and a high salt intake may have contributed to the progression of hypertension. The preference for Rubing (a firm, fresh goat milk cheese) in this population implies that lipid intake may be higher in the Yi ethnic group than in other ethnic groups in the same area.

Our study found an overall prehypertension prevalence ratio of 42.1% in men and 37.2% in women. This was higher than the ratio observed in Taiwanese adults (34%) [24] and American adults (31%) [25,26]. We found that the prehypertension prevalence ratio in the Yi ethnic group aged ≥50 was greater in men than in women, a finding consistent with other reports [24,25].

The Sino-MONICA-Beijing stroke study reported a higher incidence of stroke, particularly hemorrhagic stroke [27], in China compared with those in other countries. It was found in the systolic hypertension study in China that antihypertensive treatment targeted to achieve a SBP reduction produced a 38% reduction in stroke, which is more prevalent in China. The 2013 AHA/ACC Guidelines recommend lifestyle modification for all patients with prehypertension, including losing weight, increasing physical activity, lowering salt intake, adopting a healthy diet, and moderating alcohol consumption [23]. People with prehypertension should be informed of the seriousness of hypertension and the importance of changing any unhealthy lifestyle habits to prevent hypertension and cardiovascular disease later in life.

The rates of awareness, treatment, and control of hypertension in our study were lower than those in the Chinese population generally in 2014 (24.8 *vs*. 42.6%, 27.5 *vs.* 34.1%, and 7.2 *vs.* 9.3%, respectively) [4]. There are some possible causes to explain this phenomenon. First, the participants in our study lived in rural southwest China and had a lower degree of education, which might explain a lack in knowledge of the dangers of hypertension. Data collection in other studies included both rural and urban populations, and it is suggested that people living in urban areas have increased access to medical information. Second, most rural adults do not regularly see a doctor because of their low income, which implies that that there might be a large proportion of hypertensive patients who were not diagnosed at the early stage. Interestingly, we found that some people with higher incomes had comparatively lower levels of awareness, treatment, and control rates, regardless of possible imbalances between availability of health information, behavior formation and people’s economic status.

Our study indicates that age, BMI, cigarette smoking, alcohol intake, and family history of HBP were associated with hypertension.

Overweight and obese participants were more likely to have hypertension than people in the average weight range, while being slim was a protective factor for hypertension, findings also observed in studies in Europe [17], eastern China [5], and northeast China [16]. Odds ratios for overweight *vs.* average weight participants were 1.81 (95% CI: 1.30–2.53) in our study, 2.13 (95% CI: 1.99–2.28) in the eastern China study [5], and 2.00 (95% CI: 1.80–2.23) in the northeast China study [16].

Smoking and alcohol consumption were also identified as risk factors of HBP, results also reported in other studies on populations in eastern China [3]. Odds ratios for smoking *vs*. non-smoking participants were 1.75 (95% CI: 1.33–2.29) in this study, 1.21 (95% CI: 1.02–1.53) in the eastern China study [5], and 1.05 (95% CI: 0.89–1.25) in the northeast China study [16]. Odds ratios for alcohol consumption *vs.* non-alcohol consumption were 1.75 (95% CI: 1.36–2.25) in our study, 1.19 (95% CI: 1.07–1.31) in the eastern China study [5], and 1.40 (95% CI: 1.18–1.67) in the northeast China study [16].

A family history of HBP was another risk factor identified in the current study, which was also shown in the northeast China study [16]. The odds ratio for a family history of HBP *vs.* no family history was 3.97 (95% CI: 2.89–5.45) in the current study. Older age was also a risk factor of HBP, a finding also seen in other studies [5,16,17]. Education levels correlated to HBP. Finishing senior high school or above was identified as a protective factor for HBP, whereas finishing junior high school or below was not. This result is similar to our previous survey [22]. A possible explanation for this result might be that people with a higher education have improved access and exposure to information on HBP and how to manage it.

This study had some limitations. First, the participants may be older and unhealthier compared with the general population of rural areas because of the absence of young adults who were working in urban cities. Second, blood pressure is higher in the winter than in the summer, and daytime blood pressure is higher than nighttime. In our study, blood pressure was measured at a single visit, so its value, as well as the prevalence of hypertension based partly on the measured blood pressure, may have been overestimated. Third, this was an epidemiological study involving thousands of people. It is unrealistic to undertake expensive investigation methods (e.g., ambulatory blood pressure monitoring, aortic MRI, adrenal CT, etc.). For this reason, white-coat hypertension (where a patient exhibits elevated blood pressure in the clinical setting and not in other settings) and secondary hypertension were not excluded from the hypertensive group. In addition, target-organ damage could not be detected.

## Conclusions

We found that the prevalence rates of prehypertension and hypertension were high among Yi ethnic adults aged ≥50 in rural southwest China , and the rates of awareness, treatment, and control of hypertension were low. These results suggest that rural adults among Yi ethnic group aged 50 years or more in China are facing a challenge in the prevention and management of prehypertension and hypertension. Public health strategies involving educational and environmental interventions should be targeted at village doctors and others responsible for primary care in the Yi ethnic communities, as well as towards the Yi ethnic population. Early intervention and regular follow-ups can decrease the complications associated with hypertension. There is an urgent need to develop a hypertension education program to coordinate efforts for the detection, prevention, and treatment of hypertension in the Yi ethnic communities in China.

## Abbreviations

BMI: Body mass index
HBP: High blood pressure
ORs: Odds ratios
